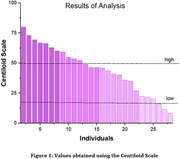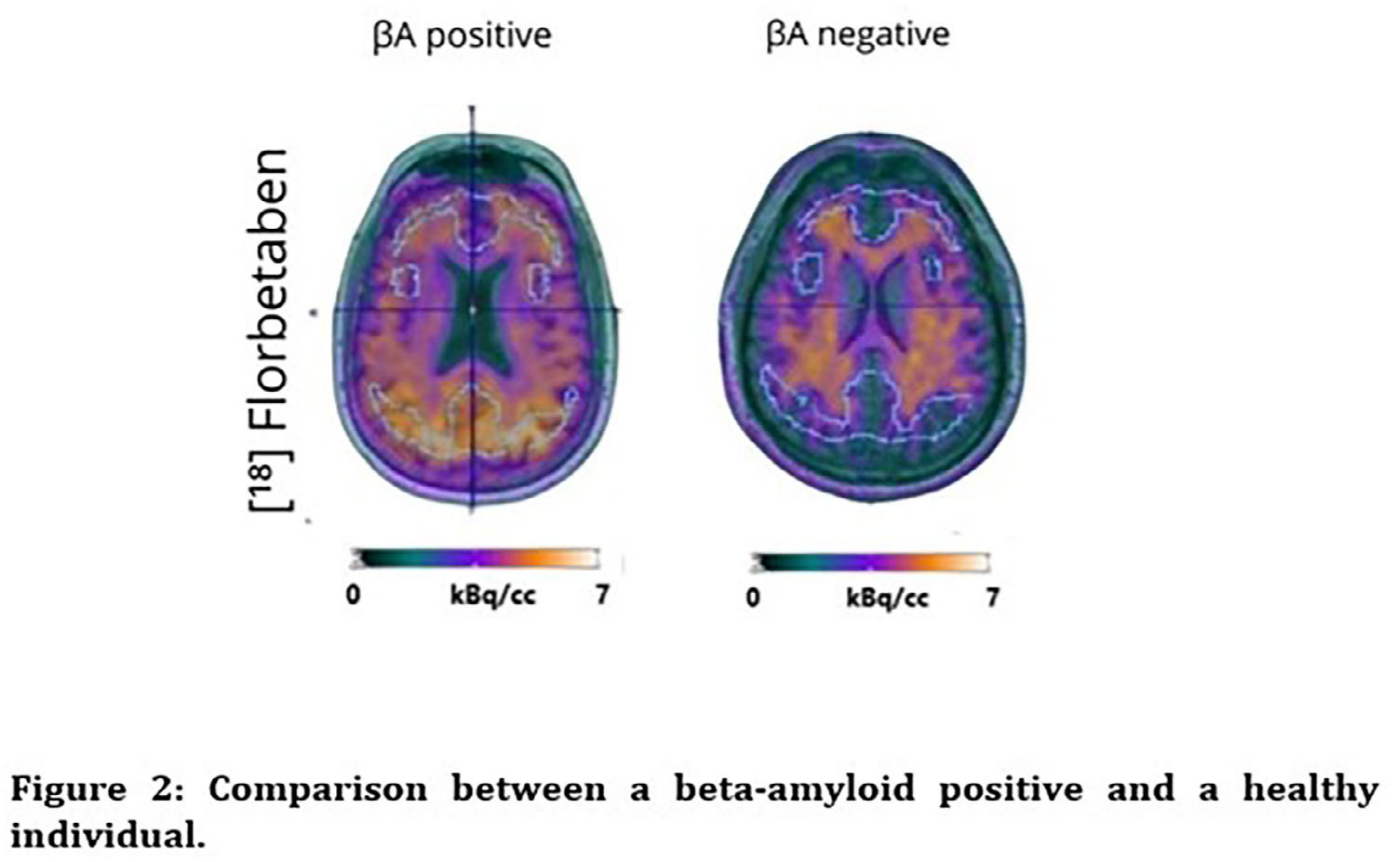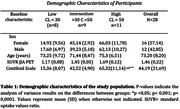# Quantification of PET amyloid [18] Florbetaben using the Centiloid Scale

**DOI:** 10.1002/alz.092829

**Published:** 2025-01-09

**Authors:** Maria Rosa Alves da Silva, Cristiano Schaffer Aguzzoli, Fabricio Nery Garrafiel, Ana Maria Marques da Silva, Lucas Porcello Schilling

**Affiliations:** ^1^ Pontifical Catholic University of Rio Grande do Sul, PORTO ALEGRE, RIO GRANDE DO SUL Brazil; ^2^ Brain Institute of Rio Grande do Sul, PUCRS, Porto Alegre, RS Brazil; ^3^ University of São Paulo Medical School, São Paulo Brazil

## Abstract

**Background:**

Alzheimer´s disease (AD) is the main neurodegenerative disorder and leading cause of dementia. AD neuropathology involves the extracellular formation of senile plaques with β‐amyloid peptide and intraneuronal tau phosphorylation, leading to neurofibrillary tangle accumulation. These can be detected by biomarkers obtained from cerebrospinal fluid or Positron Emission Tomography (PET) using specific radiotracers. Amyloid PET results can be interpreted by standard uptake values (SUVR > 1.42 positive or < 1.42 negative) or by the Centiloid scale (CL) which anchored from 0 to 100, classifying low βA uptake (<30 CL), intermediate‐high βA (>30 CL and <50 CL) and high βA (>50 CL). The aim is to analyze the distribution of CL values in [18F]Florbetaben (FBB) PET images in a sample of DA suspects.

**Method:**

A retrospective study was carried out using 28 anonymized images of FBB amyloid PET. The PET images were obtained using in the PET/CT system and the Magnetic Resonance Imaging (MRI). For this study, T1‐weighted structural images were used for co‐registration with PET images and normalization with the CL atlas. In this study, the images were processed using PMOD software with the PNEURO tool.

**Result:**

The implementation of CL required reproducing the image processing steps with the GAAIN dataset. A total of 28 participants were included in our study and Figure 1 shows the graph of this investigation. In Table 1, when analyzing the CL, the data shows a varied distribution: 8 participants below 30CL, 9 recorded values between 30CL and 50CL, and 11 presented values above 50CL. Figure 2 shows an example of a positive and negative individual. The analysis of the distribution of individuals with different stages of aging in CL allowed us to investigate how the use of different cut‐off points for the βA classification can have an impact on the diagnosis of AD pathology.

**Conclusion:**

The use of the CL is an accurate and robust method to evaluate amyloid deposition in the brain. The communication of PET scan results with the CL scale also allows normalization for any amyloid radiopharmaceuticals, making it more suitable for analysis of disease progression and response to treatment.